# Enlarged Spleen

**Published:** 1875-04

**Authors:** 


Enlarged Spleen.—Prof. Da Costa treated with success a case of leucocythemia connected with an enlarged spleen by injecting five grains of ergotin mixed in glycerine and water, every other day, for eight injections. The improvement was so rapid that he was discharged as cured after the eighth injection. The size of the spleen was diminished sensibly from day to day. The ergotin was introduced in the splenic region.—*Clinic.*

A good deal of laughter was occasioned in the French Chamber, the other day, when Dr. Testelin, in demanding a school of medicine for Lille, in addition to those proposed for Lyons, etc., declared that the number of doctors had decreased in the town that he represents, while the population had increased. The doctor appeared astonished at the amusement caused by this statement.

An Irish editor says he can see no earthly reason why women should not be allowed to become medical men.

